# Mining Daily Activity Chains from Large-Scale Mobile Phone Location Data

**DOI:** 10.1016/j.cities.2020.103013

**Published:** 2021-02

**Authors:** Ling Yin, Nan Lin, Zhiyuan Zhao

**Affiliations:** a Shenzhen Institutes of Advanced Technologies, Chinese Academy of Science, Shenzhen, China; b Academy of Digital China (Fujian), Fuzhou University, Fuzhou, China; c Key Lab of Spatial Data Mining and Information Sharing, Ministry of Education, Fuzhou, China; d National & Local Joint Engineering Research Center of Geo-spatial Information Technology, Fuzhou, China

**Keywords:** Mobile phone data, Activity chain, Activity purpose, Trajectory analysis, Data size

## Abstract

Understanding residents' daily activity chains provides critical support for various applications in transportation, public health and many other related fields. Recently, mobile phone location datasets have been suggested for mining activity patterns because of their utility and large sample sizes. Although recently machine learning-based models seem to perform well in activity purpose inference using mobile phone location data, most of these models work as black boxes. To address these challenges, this study proposes a flexible white box method to mine human activity chains from large-scale mobile phone location data by integrating both the spatial and temporal features of daily activities with varying weights. We find that the frequency distribution of major activity chain patterns agrees well with the patterns derived based on a travel survey of Shenzhen and a state-of-the-art method. Moreover, a dataset covering over 16.5% of the city population can yield a reasonable outcome of the major activity patterns. The contributions of this study not only lie in offering an effective approach to mining daily activity chains from mobile phone location data but also involve investigating the impact of different data conditions on the model performance, which make using big trajectory data more practical for domain experts.

## Highlights

• A flexible white-box method to mine activity chains from large-scale mobile phone location data is proposed
• The frequency distribution of major activity chain patterns agrees well with the patterns derived based on the travel survey.
• 16.5% of the population can yield a reasonable and robust outcome
• The spatial features play a more important role than the temporal features when inferring activity patterns

## 1 Introduction

An individual's activity chain generally refers to a series of daily activities with characteristics such as the activity order, time, location, and purpose (Bowman & Ben-Akiva, 2001; Kitamura, 1988). Understanding residents' activity chains with both spatial and temporal characteristics provides critical support for human mobility research and related applications. Particularly, during the pandemic of COVID-19, human mobility information derived from the mobile phone data and many other resources have been effectively used to evaluate the spread trend of the disease, support the epidemiological survey, generate the health code and help to make precise and customized control policies in both intra-city and inter-city scales (Aleta et al., 2020; Chinazzi et al., 2020; Zhou et al., 2020). In the transportation and urban planning, residents' activity chains offers important understanding for people's daily requirements in space, time and purposes (Bowman & Ben-Akiva, 2001). Traditional approaches to collecting activity chain information mainly depend on travel surveys, which often use a large amount of resources but lack instantaneity and continuity (Collia et al., 2003; McDonald, 2008). Currently, information and communication technologies (ICTs) ubiquitously generate individual trajectory data (e.g., geotagged social media data), thus offering the possibility of continuously perceiving human activity chains with large sample sizes at a low cost.

Mobile phone location data are commonly used trajectory datasets and have been widely used to investigate human mobility patterns. However, records in mobile phone location data usually lack direct semantic information (e.g., the activity type such as shopping or leisure). This information shortcoming prevents further applications that require more semantic information. In fact, some daily activity characteristics, such as the activity type, are hidden in a trajectory and can be inferred by several complex models through additional information derived from auxiliary datasets (Chen et al., 2016). Previous inference methods have significantly improved the usability of mobile phone location data in urban planning and other related fields.

In particular, some recently developed machine learning models seem to perform well in activity purpose inference from mobile phone location data. However, there are two limitations that prevent the use of these methods in practical applications. First, many methods involve complex training models (e.g., the neural network-based training model proposed by Yin et al. (2018)), which work as black boxes for domain experts (Bello et al., 2017). As a result, these methods usually fail to offer intuitive insights into understanding how the spatial and temporal features affect the observed residents' daily activity patterns. Second, these models require information derived from complex auxiliary datasets (e.g., city-wide household travel survey data and land use type for each fine-grained parcel, such as in a study by Diao et al. (2016)), which are not accessible for many users.

In addition to the above two limitations, there is concern about the data size that is common for many domain practitioners in the big data era: acquire as large of a data size as possible. Note that the term “size” here may indicate the sample size or the temporal ranges of a certain dataset. One important reason is that domain practitioners are not certain what data size is large enough to produce reliable results. Indeed, the large sample size is a key reason why mobile phone location data have become one of the most popular data sources in human mobility studies (Blondel et al., 2015). However, a scientific fact is that, in terms of data size, what is needed is enough for a certain research purpose, not as much as possible. Therefore, we need to investigate the impact of data size on model performance when we propose a model based on so-called big data.

To address the above issues, this study proposes a flexible white box method for mining human activity chains from large-scale mobile phone location data by integrating both the spatial and temporal features of daily activities. The robustness of the outcomes affected by the data size is then tested and discussed. The contributions of this study not only lie in proposing an effective and simple method but also involve investigating the impact of different data conditions on the model performance (e.g., the absence of spatial or temporal features and different data sizes), and these contributions make big trajectory data more practical for domain experts.

The remainder of this paper is organized as follows. In Section 2, we review related studies, including deriving human mobility patterns using mobile phone location data and inferring activity types from typical trajectory datasets. The proposed method and corresponding results are introduced in 3, 4, respectively. In Section 5, we discuss how the spatiotemporal features and the number of users affect the results, and in Section 6, we draw several conclusions.

## 2 Related work

### 2.1 Understanding human mobility patterns using mobile phone location data

Passive mobile phone location datasets based on the Cell-ID positioning method are some of the most widely used datasets collected based on mobile phone platforms in existing studies. From a spatial resolution perspective, Cell-ID positioning techniques use the locations of cell towers, which provide telecommunication services instead of accurate locations, to represent a user's current location (Ratti et al., 2006). The spatial resolutions range from hundreds of meters in downtown areas (Xu et al., 2016) to kilometers in suburban areas (Ahas et al., 2015). From a temporal resolution perspective, the average temporal intervals between adjacent records in datasets in existing studies could range from several minutes (e.g., Zhao et al., 2018) to several hours (Xu et al., 2016).

Many important studies have been conducted by using mobile phone location data. For example, mobile phone location data have been used to estimate dynamic populations on scales ranging from city-wide to nation-wide scales (Yang et al., 2016). Related outcomes have been further applied to detect social events in urban areas and to identify urban functions (Ratti et al., 2006; Tu et al., 2017). Based on these findings, decision making in analyses involving travel demand (Çolak et al., 2015), public health control (Tatem et al., 2014; Yin et al., 2019), pedestrian evacuation (Yin et al., 2019) during an emergency response, and many other related applications (Blondel et al., 2015) has been optimized.

Note that the above works need no direct semantic information from mobile phone location data. However, the outcomes of these applications could be further improved if the semantic information (e.g., the activity purposes) of mobile phone location data is available. For instance, the predictability of the next activity location could be increased and the travel patterns of mobile phone users (e.g., home-based work trips) could be better understood if the activity type could be derived from mobile phone location data. As a result, inferring activity information from mobile phone location data has become a critical topic.

### 2.2 Applications based on activity chains

Activity chains and trip chains are two closely related concepts in travel demand analyses and many other similar applications. An activity chain is an activity-oriented concept and regards each activity conducted in a local space with a specific purpose as a basic element (Esztergár-Kiss et al., 2017; Kitamura, 1988); a trip chain considers the trips between different activities and is a trip-oriented concept (2001 National Household Travel Survey of the USA). In previous studies, these two concepts have been used according to specific needs (e.g., activity-oriented purposes or trip-oriented purposes). This study uses an activity chain since we mainly focus on the inference of the activity purposes embedded in mobile phone location data.

Activity chains were introduced as activity-based models to optimize travel demand analyses in transportation planning fields (Bowman & Ben-Akiva, 2001). The four-step model has long been the method to estimate travel demand in transportation planning (Martin & McGuckin, 1998). However, this model is a trip-based model and does not consider the connections and constraints among each individual's trips or those for the trips of the members of a household (Bowman & Ben-Akiva, 2001; Yin et al., 2018). To address this limitation, transportation planners introduced an activity-based model to better estimate travel frequencies and to determine the travel mode (Bowman & Ben-Akiva, 2001). The activity purpose is an important feature in an activity chain. With this feature, an activity chain enables a fine-grained description of people's daily activities, such as the temporal information and the linkages between different activities. These activity chain-related concepts also benefit many other related applications, including agent-based simulation models in epidemic control applications (Saravanan et al., 2013) and gender inequality in activity patterns (Meloni et al., 2009). However, obtaining peoples' activity chains is not easy.

### 2.3 Obtaining activity chain information

Activity chains were traditionally obtained from travel survey reports. However, conducting a city-wide household travel survey requires extensive resources. For instance, it cost approximately four million dollars to complete a 2001 travel survey containing approximately 40,000 households in the state of California (Hartgen & San Jose, 2009). Therefore, even developed cities and countries conduct travel surveys every 5 or 10 years. As a result, travel survey approaches usually lack instantaneity and continuity (Collia et al., 2003; McDonald, 2008). Recent developments in mobile communication technologies make it possible to track an individual's daily activity locations. These datasets usually lack direct semantic labels about the corresponding activities due to privacy concerns or limitations in the collection methods. However, some activity semantics are embedded in the trajectories and could be inferred. For example, Gong et al., (2016) proposed a Bayes rules-based method to infer trip purposes from taxi trajectory data by considering both spatial and temporal constraints. Mobile phone location data are common in these datasets, but effectively inferring activity chains from mobile phone location data remains a challenge for domain experts.

Methods of inferring activity chains from mobile phone location data can be divided into two types. The first type is regression-based models. Diao et al. (2016) built a multinomial logit regression model based on travel survey data to predict the semantic labels (e.g., home, work, and leisure) for mobile phone location data by considering the temporal distributions of different activities, weather factors and many other related factors. The second type is mainly based on hidden Markov network (HMN) models. Widhalm et al. (2015) proposed a semisupervised model to infer the activity chains for two mobile phone location datasets from Boston and Vienna. Related findings are consistent with the results derived from travel surveys. Yin et al. (2018) proposed an improved HMN model that considers the transition patterns between different activities to infer activity chains for regular commuters.

The above models suffer from several limitations. First, because complex iterative procedures are often involved to train the models, these models work as black boxes, which are highly complex and can hardly provide explicit explanations for the effects of input features. Examples include the training process based on an expectation maximization (EM) approach during parameter estimation in the input-output hidden Markov model in Yin et al. (2018) and the parameter optimization of the clique potential functions in the relational Markov network model in Widhalm et al. (2015). Second, auxiliary datasets that are not accessible to many users are required in these methods. For instance, travel surveys (Diao et al., 2016) that can be used to generate transition probabilities between activities are available only for the corresponding transportation agencies. Similarly, fine-grained land use data and building data (Widhalm et al., 2015) are seldom accessible to common users. If there is no alternative data source, related studies can hardly be applied due to the data availability issue. Third, these methods usually involve a large number of records. For instance, mobile phone location datasets often contain millions of users and billions of records (Cao et al., 2019; Deville et al., 2014). Even an auxiliary dataset, such as travel surveys, could involve tens of thousands of households or hundreds of thousands of users. Determining how to effectively handle these data is also a challenge for some domain experts who might be unfamiliar with complex computer programming of complex mathematical modeling.

In addition to the above challenges, stop identification is a primary step for these methods. However, there are some noisy records in mobile phone location data that may affect the stop identification results. The ping-pong phenomenon is a typical phenomenon that emerges when the current cell tower switches because the change in signal strength of the nearby cell tower is not caused by travel behavior but by the statuses (e.g., battery, antenna orientation) of the mobile phone and the surrounding environment (Iovan et al., 2013). This phenomenon causes quick location changes between nearby cell towers. As a result, there are some location oscillation and drift patterns in raw mobile phone location data. Detecting and removing these noisy records from a dataset is critical for improving the outcomes of the inferred activity chains (Horn et al., 2014; Zhao et al., 2018).

## 3 Methods

**Fig. 1:**
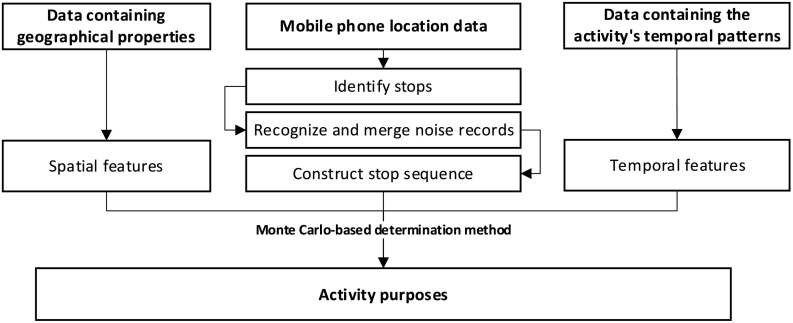
The framework of the activity purpose inference algorithm.

Fig. 1 shows the framework of the proposed approach for mining activity chains from mobile phone location data. We first design an incremental clustering algorithm based on a time-sliding window to detect individual stops from raw mobile phone location data. The noise in the raw dataset, including the oscillation records and drift records, is detected and merged in this phase to obtain more reliable stop identification results. Then, we propose a flexible inference algorithm to infer an activity purpose for each stop by considering both the spatial and temporal features. A Monte Carlo-based method is used to determine the final activity purposes based on the integration of the spatial and temporal features.

### 3.1 Definitions

**Fig. 2:**
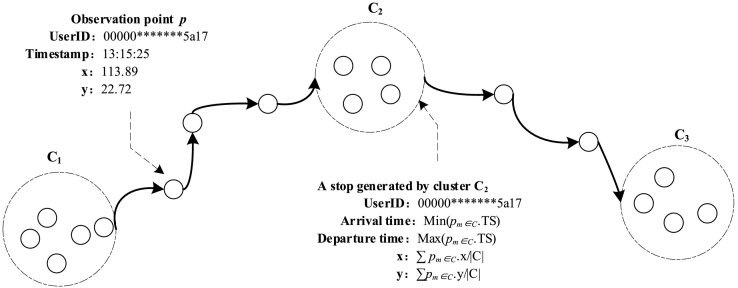
Stops and pass-by points in a trajectory.

Stop identification is a basic step, as shown in Fig. 1. The pass-by points and stops are illustrated in Fig. 2. To better indicate the stop identification method, several related concepts are required.

Definition 1: Record

A record is an observation point *p* = < *userID*, *TS*, *x*, *y*> that denotes a raw mobile phone location record with a user's anonymous ID (*userID*), the timestamp (*TS*), and the location of the corresponding cell tower located by *x*, *y*.

Definition 2: Record cluster

A record cluster *C* = {*p*_1_, *p*_2_, …, *p*_*m*_, …, *p*_*M*_}, ∀ *m* ∈ Z^+^, 1 ≤ *m* ≤ *M* denotes a cluster of several observation points that satisfy certain spatiotemporal constraints (e.g., a spatial extent constraint), and *M* denotes the number of observation points in the cluster. The records in *C* are sorted by the timestamp, which implies that *p*_*i*_. *TS* < *p*_*j*_. *TS*, where 1 ≤ *i* < *j* ≤ *M*.

Definition 3: Activity purpose

An activity purpose refers to what an individual will do during a stop behavior. This study classifies residents' trip purposes into *K* types and uses *I* = {*s*_1_, *s*_2_, …, *s*_*k*_, …, *s*_*K*_}, ∀ *k* ∈ *Z*^+^, 1 ≤ *k* ≤ *K* to represent the activity purposes set.

Five activity purposes are predefined, namely, “Home (H)”, “Work (W)”, “School (S)”, “Leisure (L)” and “Other (O)”. These five activity purposes are selected by considering two reasons: (1) referring to the previous work of Widhalm et al. (2015) to make the outcomes more comparable; (2) the 11 original activity purposes in the travel survey data of the study area are too fine, such that some of them can hardly be distinguished by the spatial and temporal features. For example, “Go to work”, “Go to business” and “Back to work” have similar semantic meanings. We regard different activity types with similar semantic meanings as one general activity type in this study (see Table 2 in Section 4.1 for detailed information).

Definition 4: Stop

A stop *st* = < *userID*, *x*, *y*, *arrT*, *depT*, *s*_*k*_> denotes a daily behavior in which an individual stays in a local area (defined by a distance threshold *ε*) for a certain period (defined by a time threshold *τ*). If a record cluster *Cn* satisfies the constraint defined by Eq. (1), that cluster will be identified as a stop. The arrival and departure times are the timestamps of the first and last records in cluster *Cn*, respectively. *s*_*k*_ indicates the specific activity purpose of the stop (Fig. 2).

(1) $$\mathrm{Distance}\left(p_{i\in C_{n}},p_{m}\right)\leq \varepsilon \;\mathrm{and}\;p_{M}.\mathrm{TS}-p_{1}.\mathrm{TS}\geq \tau$$

Definition 5: Stop sequence

A stop sequence consists of a series of stops belonging to the same person and is sorted by timestamp. This can be described by *Trj* = {*st*_1_, *st*_2_, …, *st*_*n*_, …, *st*_*N*_}, ∀ *n* ∈ Z^+^, 1 ≤ *n* ≤ *N*, where *st*_*n*_ is the *n*^*th*^ stop and *N* denotes the number of detected stops. Note that the activity purpose of *st*_*n*_ is null in this phase.

Definition 6: Activity chain

If the activity purpose at each stop in *Trj* is determined, this stop chain can be transformed into an activity chain. The sequence of activity purposes derived from the stop chain could be used to represent the activity chain pattern. For example, “*Home-Work-Home*” could be used to describe the activity chain of an individual going to work from home in the morning and going back home after work in the afternoon.

### 3.2 Detecting oscillation and drift records

To reduce the influence of location oscillation and location drift noise on the results, we propose two rule-based approaches to detect corresponding noise records based on location change patterns.

(1) Recognizing oscillation records

**Fig. 3:**
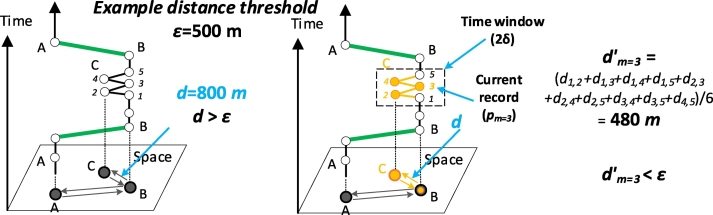
The rule for detecting oscillating points. *d*_*m*=3_′ is the average distance between pairs of points during the time window defined by a width of 2*δ* and centered by the timestamp of *p*_*m*=3_. The oscillating records and corresponding features are marked in orange.

Oscillating points are usually shown to be reciprocated among several adjacent cell towers over a period (e.g., the records at location C in Fig. 3). Due to the oscillation phenomenon, the average distance between any two points (*d*_*m*=3_′ in Fig. 3b) in the trajectory segments is not as large as the displacement distance (*d* in Fig. 3a).

This study defines a sliding time window by the timestamp of point *p*_*m*_ as the center of the window and a window width of 2*δ* to extract adjacent records and calculate the average distance between them. This study recognizes *p*_*m*_ as an oscillating point if the average distance between any two points in the time window is smaller than the distance threshold *ε*. Eq. (2) shows the formalized expression of this rule:

(2) $$d_{m}^{\prime }\leq \varepsilon$$

where *m* is the identification of the record *p*_*m*_ and *d*_*m*_′ is the average distance between pairs of points in the time window defined by a width of 2*δ* and centered by the timestamp of the record *p*_*m*_. Fig. 3 provides an example of how this rule works to detect the oscillation point *p*_*m*=3_.

(2) Recognizing drift records

**Fig. 4:**
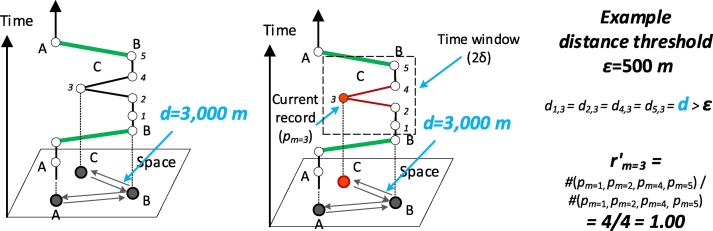
The rule for detecting drift records. *r*_*m*_′ indicates the proportion of the number of points that are significantly far from the current point to the number of points in the time window. *p*_*m*=3_ (marked in red) is a drift record since the distances between all four records in the time window and the center record *p*_*m*=3_ are larger than the distance threshold and *r*_*m*=3_′ is 1.00. Comparatively, *r*_*m*=2_′=0.25 if *p*_*m*=2_ is the current record, and this value is significantly smaller than 1.00. (For interpretation of the references to color in this figure legend, the reader is referred to the web version of this article.)

Drift points are usually far away from the adjacent points (e.g., the record at location C in Fig. 4). The presented rule introduces a voting mechanism by counting the number of points whose distances from the observation point *p*_*m*_ are larger than the distance threshold *ε*: #[*p*_*i*_ : *Distance*(*p*_*m*_, *p*_*i*_) > *ε*]. Specifically, this rule calculates the distance between each adjacent point of *p*_*m*_ extracted by the time-sliding window and judges whether the distance is larger than the distance threshold *ε*. If the proportion *r*_*m*_′ of #[*p*_*i*_ : *Distance*(*p*_*m*_, *p*_*i*_) > *ε*] to the number of records in the time window #(*p*_*j*_) is larger than the voting threshold *ξ*, the rule treats *p*_*m*_ as a drift point. The formalized expression of this rule is shown in Eqs. (3), (4):

(3) $$r_{m}^{\prime }>\xi$$

(4) $$r_{m}^{\prime }=\#\left[p_{i}:\mathrm{Distance}\left(p_{m},p_{i}\right)>\varepsilon \right]/\#\left(p_{j}\right)$$

$$s.t.i\neq m;p_{m}.\mathrm{TS}\leq p_{i}.\mathrm{TS},p_{j}.\mathrm{TS}\leq p_{m}.\mathrm{TS}+\delta ;0\leq \xi \leq 1$$

Given a series of adjacent points to *p*_*m*_, the voting threshold *ξ* is essentially the confidence with which the rule identifies *p*_*m*_ as a drift point. A *ξ* value close to 1 indicates strong confidence; otherwise, low confidence is indicated.

(3) Identifying stops and merging noise records

We first use Eq. (1) to identify *stops* and then recognize the oscillation and drift records by the above approaches for the rest of the trajectory. If there is no other record between a stop and an oscillation or drift record, the record is added to the stop. The stop sequence is constructed based on the modified stop identification results for each user.

Notably, the methods of recognizing the oscillation and drift records are rule-based approaches. These rules are defined based on typical characteristics for distinguishing noise records and normal daily activity patterns. For example, the rule to detect the oscillation records is designed to catch frequent and repeated movements among nearby locations during a relatively short period. The rule to detect the drift records is designed to recognize sudden and long movements followed by a short-lasting activity. These two patterns are unusual in people's daily lives. With strict parameter setting, these two rules can detect the corresponding noise records effectively.

### 3.3 Inferring activity purposes based on spatial and temporal features

(1) Calculating activity purpose probabilities based on spatial features

People's daily activities are closely related to the spatial features (e.g., land use types and building types) around their activity locations (Yang et al., 2019). The relationship between daily activities and geographical properties can be used to support the inference of one of them if the other is given. To infer activity patterns based on geographical properties, a basic assumption is used in a study by Widhalm et al. (2015), as well as in this study: a person engages in activities that are closely related to the corresponding spatial features.

First, the linkage between spatial features and activity purposes must be built. Spatial features are usually described as enumerated types (e.g., building types and land use types).

**Fig. 5:**
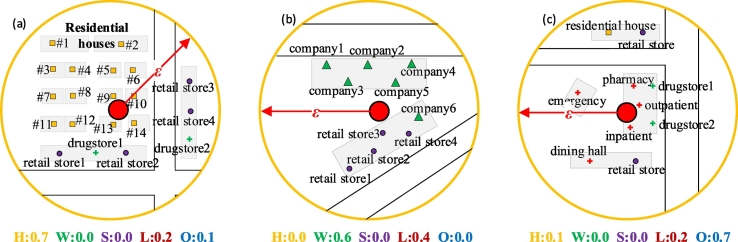
The rule for determining activity purposes based on spatial features measured by proportions and using POI data as an example: (a) indicates a typical residential area, (b) indicates an area with a large office building, and (c) indicates an area around a hospital. The text below each circle indicates the proportions of the types of POIs. Note that residential houses and companies relate to “Home (H)” and “Work (W)” activities, respectively, while retail stores and drugstores correspond to “Leisure (L)” and “Other (O)” activities, respectively, in this figure.

Then, given a stop *st*_*n*_, ***Α*** = {*α*_1_, *α*_2_, …, *α*_*k*_, …, *α*_*K*_} depicts the probability vector corresponding to different activity purposes. *α*_*k*_ is the probability that the stop corresponds to activity purpose *s*_*k*_, and this probability can be quantified by the proportion of spatial features related to activity purpose *s*_*k*_ in a local area defined by the center of this stop and the distance threshold *ε*, as shown in Eq. (5):

$$\alpha_{k}=\frac{\sum_{l=1}^{L}f\_{\mathrm{sf}}_{k,l}}{\sum_{k=1}^{K}\sum_{l=1}^{L}f\_{\mathrm{sf}}_{k,l}}$$

(5) $$s.t.\mathrm{Distance}\left[{\mathrm{sf}}_{h},\left({\mathrm{st}}_{n}.x,{\mathrm{st}}_{n}.y\right)\right]\leq \varepsilon$$

where *f*_*sf*_*k*, *l*_ represents the frequency of the spatial features belonging to enumerated type *l* that is related to activity purpose *s*_*k*_, *L* is the number of enumerated types related to *s*_*k*_, and *sf*_*h*_ indicates each spatial feature. Fig. 5 provides three typical scenarios of how Eq. (5) works by using point of interest (POI) data as an example.

(2) Calculating activity purpose probabilities based on temporal features

**Fig. 6:**
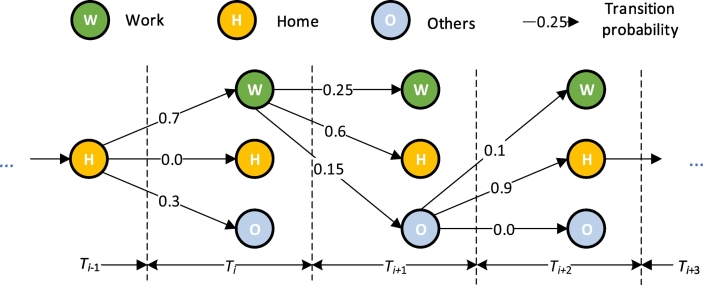
The rule for determining the current activity purpose based on the transition probabilities derived from the temporal features. Note that only three types of activities are listed as examples here for simplicity.

Residents' daily schedules are often constrained by social rules, thus forming rhythmic daily behaviors. The transition patterns between different types of activity purposes (e.g., Fig. 6) can depict such rules. The transition patterns can be extracted from auxiliary data, such as residents' travel survey data or social network data.

Given a dataset that includes activity tracking information of the sampled individuals, the probability of an activity type *p*(*s*_*k*_)_*T*_ during a time window *T* and the transition probabilities *p*(*s*_*k*_| *s*_*i*_)_*T*_ between different activity types during the time window *T* and the subsequent time window *T* + 1 can be derived as follows:

(6) $$p{\left(s_{k}\right)}_{T}=\frac{f{\left(s_{k}\right)}_{T}}{\sum_{k=1}^{K}f{\left(s_{k}\right)}_{T}}$$

(7) $$p{\left(s_{k1}|s_{k2}\right)}_{T}=\frac{f{\left(s_{k1}|s_{k2}\right)}_{T}}{\sum_{i=1}^{K}f{\left(s_{k1}|s_{i}\right)}_{T}}$$

where *f*(*s*_*k*_)_*T*_ denotes the number of individuals participating in an activity with purpose *s*_*k*_ during a time window *T*; if there are two or more activity types during *T*, the activity type with the longest duration is assigned to *s*_*k*_ (e.g., “go to work” and “sends/pick up” may happen from 8:00–9:00 am, but only “go to work” will be assigned if it lasts longer). *f*(*s*_*k*1_| *s*_*k*2_)_*T*_ denotes the frequency of transitions from an activity purpose *s*_*k*2_ in a time window *T* to another activity purpose *s*_*k*1_ during the time window *T* + 1, where *k*1, *k*2, *i* ∈ {1, 2, …, *K*}. Given a stop *st*_*n*_, this study uses Eqs. (6), (7), (8) to determine the probability vector ***B*** = {*β*_1_, *β*_2_, …, *β*_*k*_, …, *β*_*K*_} corresponding to different activity purposes based on temporal features. *β*_*k*_ is the probability of an activity purpose *s*_*k*_ and can be calculated by Eq. (8):

(8) $$\beta_{k}=\left\{\begin{array}{c} p{\left(s_{k}\right)}_{T\_\mathrm{arr}}\\ p{\left(s_{k}|{\mathrm{st}}_{n-1}.s\right)}_{T\_\mathrm{arr}} \end{array}\right)\;\begin{array}{c} n=1\\ n>1 \end{array}$$

Specifically, given a *stop sequence*, the probability of a specific activity purpose for the first stop is measured by the probability of each type of activity purpose during the time window containing the arrival time of the stop *T*_*arr*. Otherwise, *β*_*k*_ will be estimated by the transition probability from the activity purpose of the last stop. Fig. 6 indicates how Eq. (8) works. The arrows and numbers indicate the transition probabilities between corresponding activity purposes.

(3) Inferring activity purposes based on the combination of spatial and temporal features

To integrate spatial and temporal features together and explicitly represent their own effects on the inference of activity purposes, we adopted a straightforward model that linearly combines these two features. Note that since it is not easy to collect or access a “ground truth” dataset of individual samples with a fine spatiotemporal resolution to represent the entire population of mobile phone users, a supervised linear model such as linear regression is excluded. Therefore, we developed a weighted linear model, where the two weights can explicitly represent the effects of spatial and temporal features on activity type inference.

Specifically, a weighting parameter *λ* ∈ [0, 1] is used to flexibly combine the inferred results of activity purposes from both the spatial features and the temporal features. We denote *γ*_*k*_ to represent the combined probability that an individual will perform an activity of type *k* during a certain stop. An intuitive and straightforward method defined by Eq. (9) is adopted to combine the probabilities derived from the two types of features:

(9) $$\gamma_{k}=\lambda \alpha_{k}+\left(1-\lambda \right)\beta_{k}$$

According to Eq. (9), *λ* = 0 indicates that only the temporal features work to generate the combined probability, while *λ* = 1.0 indicates that only the spatial features work. In contrast, *λ* = 0.5 indicates that the spatial and temporal features play equally important roles, and a larger *λ* indicates that spatial features account for greater importance in generating the combined probability. Like ***A*** and ***B***, we denote ***Γ*** = {*γ*_1_, *γ*_2_, …, *γ*_*k*_, …, *γ*_*K*_} to represent the set of probabilities of *K* types, where ∑*γ*_*k*_ = 1.

A Monte Carlo simulation method is adopted to determine the specific activity purpose for each stop based on its activity purpose probability distribution *Γ*. In this setting, an activity purpose with a relatively small transition probability might be chosen, which is one of the reasons why the activity purpose “Other (O)”, whose transition probability is relatively low, is chosen during the time window *T*_*i*+1_ in Fig. 6.

### 3.4 Measuring the effectiveness using the Kullback-Leibler (KL) divergence based on travel survey data

Since the ground truth of activity chains derived from mobile phone location data is not available, a city-wide travel survey can be treated as a benchmark to evaluate the mining results.

We use the Kullback-Leibler (KL) divergence to measure the differences between the proportions of typical activity chain patterns derived from mobile phone location data and travel survey data. The KL divergence is well known as the relative entropy between an approximated probability distribution and a benchmark probability distribution (Kullback, 1997). The KL divergence is defined as follows:

(10) $$D_{\mathrm{KL}}\left(g,h\right)=\sum_{x\in X}g\left(x\right)*\log\left(\frac{g\left(x\right)}{h\left(x\right)}\right)$$

Here, *D*_*KL*_(*g*, *h*) is the KL divergence value, which measures how close the approximated distribution *h*(*x*) is to the benchmark distribution *g*(*x*). This is a commonly used method in statistics as a measure of the similarity between different probability distributions (Olsen & Dharanipragada, 2003). Larger divergences indicate larger differences between the two probability distributions.

## 4 Results

### 4.1 Data sources

**Fig. 7:**
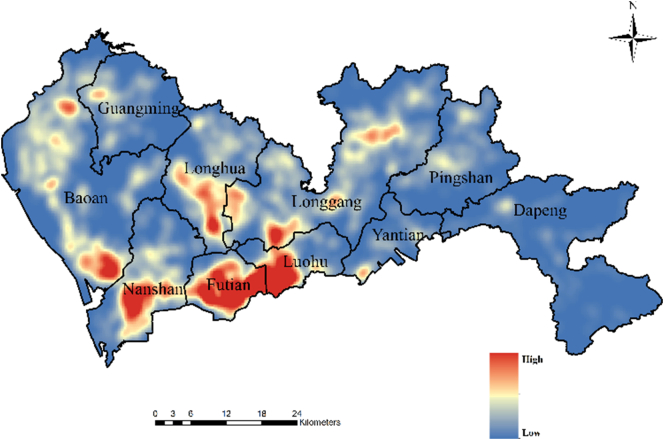
Density distribution of the cell towers.

The mobile phone location dataset used in this study includes approximately 5.8 million mobile phone users in Shenzhen, China, and approximately 6000 cell towers; the average distance between cell towers is 325 m (see Fig. 7). This dataset was collected regularly with a nearly fixed temporal sampling interval of 1 h on a weekday in March 2012. Therefore, there is at least one record for each user at every hour during the day (more detailed information about the temporal intervals can be found in Fig. A.3 in Appendix A). All the users are anonymous, and example records are shown in Table 1. In terms of the representativeness of the dataset, Xu et al. (2016) found that the Pearson coefficient between the populations by administrative districts derived from the same mobile phone location data as in this study and recent census data is 0.99 at the district level (10 districts in 2012). The coefficient decreases slightly to 0.95 at the sub-district level (55 sub-district units in 2012). These findings indicate that this dataset has a good representativeness of the population.

**Table 1:** Format of the mobile phone location data.

Anonymous ID	Time stamp	Longitude	Latitude
460********9251	2013-**-**T 0:01:23.000Z	114.****	22.****
460********2565	2013-**-**T 07:07:55.000Z	114.****	22.****
460********3757	2013-**-**T 10:14:11.000Z	114.****	22.****

**Fig. 8:**
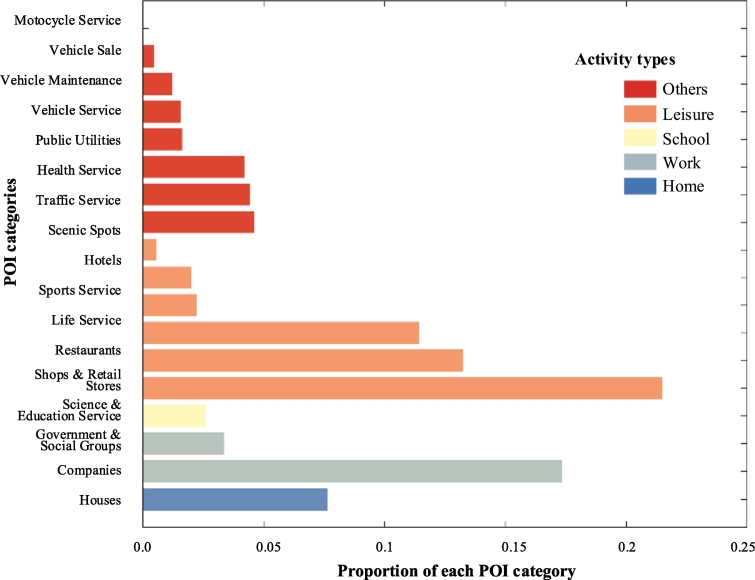
Mapping between POI categories and the predefined activity purposes. The proportion of each POI category is shown by the horizonal axis.

A POI dataset and a travel survey dataset are used to depict the spatial and temporal features in this study, respectively. First, the POI dataset is from 2013 and is obtained from *www.amap.com*, which is one of the most popular map websites in China. This dataset includes approximately 150,000 records with locations and covers 17 categories (see the vertical axis of Fig. 8). The relationships between the POI categories and the predefined activity purposes are shown in Fig. 8.

**Fig. A.1:**
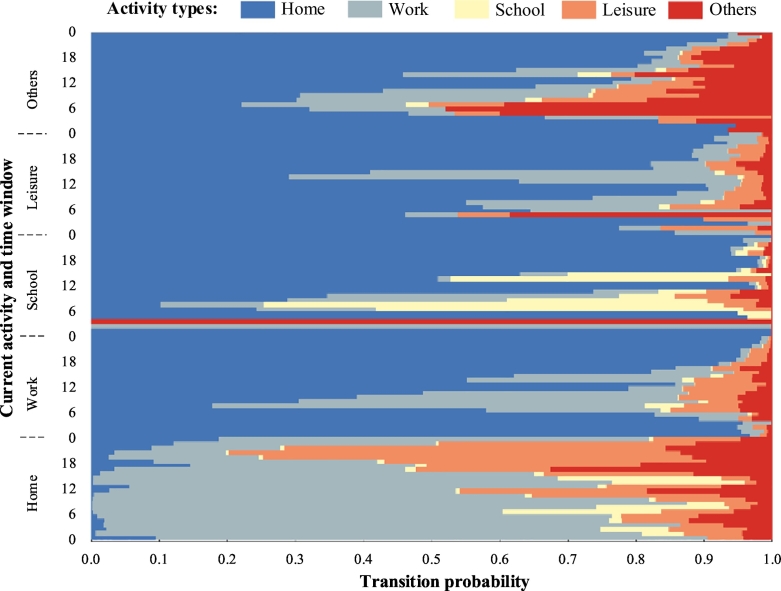
The hourly activity transition probability in Shenzhen was derived from the travel survey data collected in 2010. The hourly color vector indicates the transition probabilities from the corresponding previous activity type in the vertical axis to different activity types characterized by different colors.

The travel survey dataset was collected by the Urban Planning, Land and Resources Commission of Shenzhen municipality in October 2010. This dataset includes 11 types of trip purposes for approximately 190,000 residents. We map these 11 trip purposes to the 5 predefined activity purposes according to the semantic information. The specific rules are shown in Table 2. The hourly transition probabilities between different activity purposes can then be calculated based on the travel survey dataset (see Fig. A.1 in Appendix A).

**Table 2:** Mapping table of the trip purposes in the travel survey data to the 5 activity purposes.

Predefined activity purposes	Original trip purposes in travel survey data
“Home” (H)	“Go home”
“Work” (W)	“Go to work”, “Go to business”, “Back to work”
“School” (S)	“Go to school”
“Leisure activities” (L)	“Go shopping”, “Entertainment”
“Other activities” (O)	“Visit”, “Go to hospital”, “Pick up”, “Other”

Notably, both the travel survey dataset and the POI dataset are auxiliary datasets. However, they have different data accessibility conditions. Specifically, the POI dataset is usually accessible to many people, while the travel survey dataset is not. This is an important reason why we propose a flexible method with a weight parameter to combine the features derived from these two datasets in different conditions. By setting a corresponding weight parameter, the proposed method can still work when either of the two auxiliary datasets is available.

### 4.2 Parameter setting

First, the spatial threshold *ε* is a critical parameter in detecting oscillating records, detecting drift records and identifying stops. There is no uniform standard default value for this parameter. Two issues are usually considered: (1) the normal spatial extent of daily activities is often reflected by the definition of a trip according to the distance dimension (e.g., larger than 500 m), and (2) the spatial resolutions of the mobile phone location data are often quantified by the distances between cell towers. This study also considers these factors and sets the spatial threshold *ε* to 500 m.

Second, the temporal threshold *τ* is a threshold for determining whether a user stays in a local area long enough and whether corresponding records could be identified as a stop. This parameter is determined according to the specific research purpose and the temporal intervals of the dataset. In terms of the data conditions, the temporal resolution of the mobile phone location data is approximately 1 h. In terms of the research purpose, the selected activity purposes in this study have long duration times in people's daily lives (e.g., longer than 1 h). Therefore, we set *τ* to 1 h to identify stops and help to detect the noise records.

Third, the other parameters for detecting oscillating records, detecting drift records and inferring activity purposes are set with the following considerations. The time window width *δ* described in Section 3.2 for detecting oscillation and drift records is set to 1 h considering the temporal resolution of the dataset. We set the ratio threshold *ξ* to 1.0 to obtain rigorous detection results with high confidence in detecting drift records by Eq. (3). In addition, the default weighting coefficient *λ* is set to 0.5 with a preliminary assumption that the spatial and temporal features equally contribute to inferring an activity purpose. In the Discussion section, we discuss the impact of the value of *λ* on the outcomes.

### 4.3 Statistical patterns of the derived activity chains

Using the parameters set in the last section, we detect the stops of each user in the mobile phone location data, and the corresponding activity purpose of each stop is inferred. We use the activity purpose sequence to represent the activity chain pattern. Since the individuals with no daily travel are not included in the travel survey dataset, corresponding users in the mobile phone dataset who only have only one identified stop are excluded during the following comparative analysis. As a result, the top 8 most frequent activity chain patterns derived from the inferred activity purposes and from the travel surveys are listed in Table 3. To better compare the effectiveness of the proposed method, we implement the approach of Widhalm et al. (2015) because of the similar theoretical assumptions and data conditions of inferring activity purposes based on temporal features relying on travel surveys and spatial features derived from related auxiliary datasets (i.e., a land use dataset vs. the POI dataset). The outcomes of the different approaches are shown in the table.

**Table 3:** Comparison of activity pattern distributions between the mobile phone location data and the travel survey data.

Activity patterns	Mobile phone location data		Travel survey data
	Proposed method	Widhalm et al. (2015)	
HWH	67.93%	57.56%	56.98%
HWHWH	6.51%	2.02%	9.86%
HSH	6.58%	18.88%	7.23%
HLH	4.87%	0.00%	5.49%
HSHSH	0.51%	0.34%	3.12%
HWLH	0.79%	0.00%	1.80%
HWHLH	0.16%	0.00%	1.69%
HOH	2.71%	15.18%	1.58%
Other patterns	9.94%	6.02%	12.25%

Table 3 presents several interesting results. First, the overall distribution of typical activity patterns derived from the mobile phone location data is consistent with the results from the travel survey data. The values of Spearman's correlation coefficient between the travel survey results and the results derived from the above two methods are 0.81 (p-value = 0.02) and 0.51 (p-value = 0.19), respectively. The outcomes of our proposed method are more consistent than those of the state-of-the-art method proposed by Widhalm et al. (2015)*.* Specifically, the state-of-the-art method performs well in inferring the “HWH” pattern, which is the main daily activity pattern. However, it fails to capture the activity patterns that contain “Leisure” from the mobile phone location dataset.

Two potential reasons may underlie this phenomenon: (1) the approach in Widhalm et al. (2015) does not consider the transition probabilities between different activities in different time windows, which is very important in capturing these short-duration and easily ignored activities. For instance, the transition probability from “Work” to “Leisure” (e.g., having lunch) tends to have a relatively high value at noon. (2) The Monte Carlo simulation method may choose some activity purposes even though the probability of the target activity purpose is not the highest.

**Fig. A.2:**
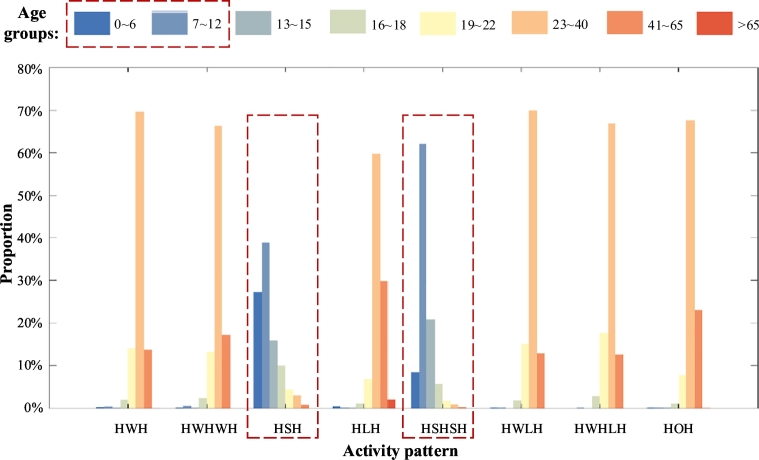
Age distribution of residents with 8 typical activity patterns in the travel survey data. Children younger than 12 years old contributed the most to the activity patterns containing “School”.

**Fig. A.3:**
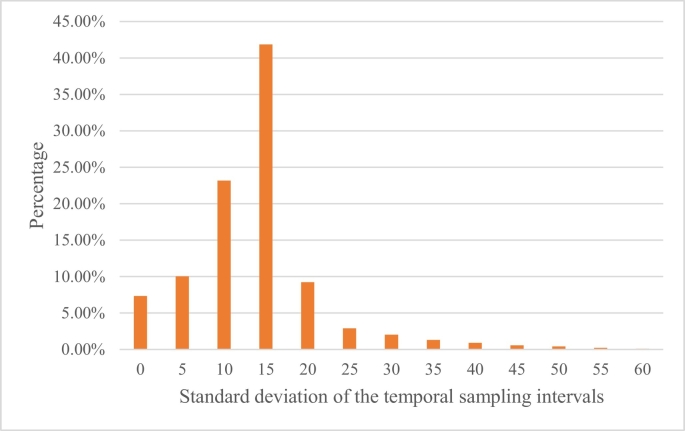
The distribution of the standard deviation of the temporal intervals in the dataset.

Second, the proposed method tends to underestimate the proportions of activity patterns containing “School”. Sampling bias tends to be the main reason: the number of school children is greatly underestimated since they are usually prevented from having mobile phones only at school. The activity patterns containing “School” in the travel survey data are mainly contributed by children younger than 12 years old (see Fig. A.2 in Appendix A).

Third, “Leisure activities”-related activity patterns that derived from the mobile phone location data are underestimated. One potential reason is that these activities are discretionary acts, which tend to take shorter times than those obligatory acts such as work or sleep (Golledge & Stimson, 1997). These activities are easier neglected in a mobile phone location dataset when its temporal sampling is sparse (e.g. one hour) (Zhao et al., 2019). Consequently, using the analysis results should be very careful in related applications, especially for official or critical policy-makings. For example, *contact rate* (*McGraw-Hill Concise Dictionary of Modern Medicine*, Retrieved September 12 2020) is the rate that at which susceptibles meet infecteds in *Epidemiology*. It is a key parameter in the compartmental models in epidemic control such as SIR, SEIR models etc. (Allen, 2008). This parameter changes in different situations. Many leisure activities (e.g., watch a movie in a cinema or go to a stadium to watch a football match) involve higher contact rates than those regular activities such as go to work or go to school. Neglect of these acts tends to result in stronger underestimation of the epidemic degrees of related infectious diseases (e.g., COVID-19). In terms of the transportation planning field, travel demand analysis based on the mobile phone location data tends to underestimate the travel demand involves the “Leisure activities”. Corresponding transportation service supplies will be inadequate and lead to negative impacts on the development of related areas.

### 4.4 Temporal distributions of the derived activity chains

Several common patterns can be observed for the activity patterns derived from the mobile phone location dataset and the travel survey dataset. For instance, “Home” and “Work” are the two main activity purposes, and their activity proportions shift from one to the other at the peak morning hours and the peak evening hours. The proportions of “Other” exhibit two peaks, one of which is during the night.

**Fig. 9:**
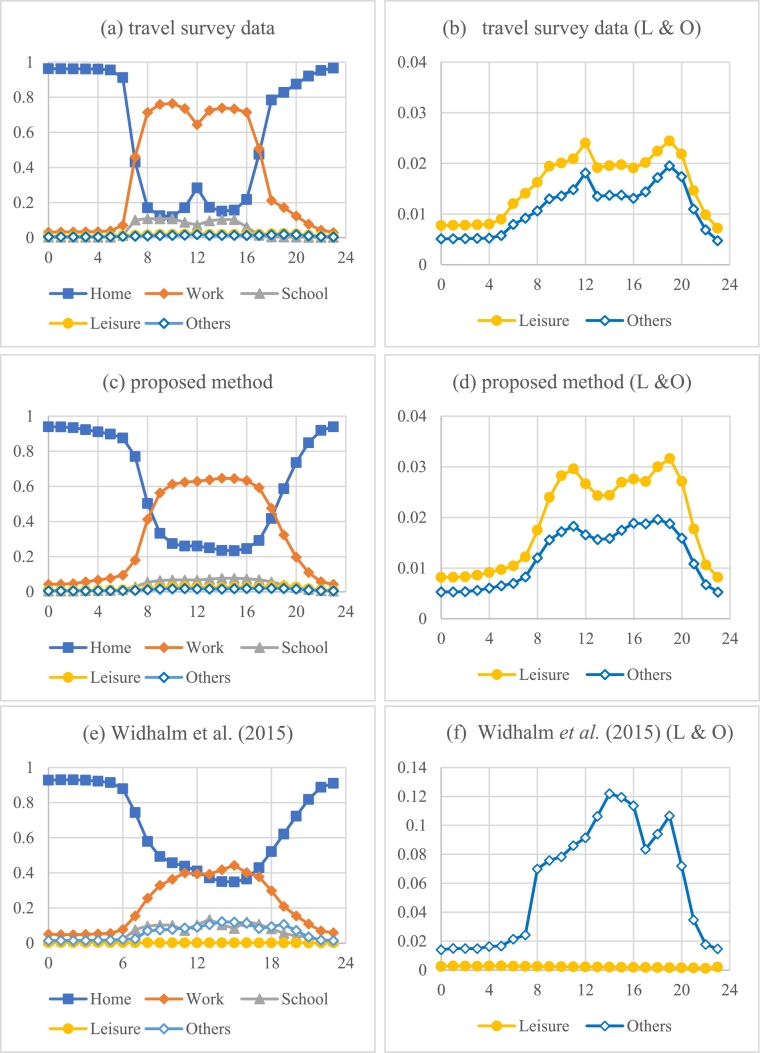
Activity distributions at different times. The vertical axis shows the probability of each activity, and the horizontal axis shows the hours of the day. Note that the figures on the right side are enlarged versions on the vertical axis of the “Leisure” and “Other” types in the corresponding figures on the left side.

However, Fig. 9 also shows some typical differences. First, the mobile phone location dataset significantly underestimates the patterns from the travel survey data containing lunch breaks at home during work hours. One of the important reasons for this underestimation is because lunch breaks are relatively short (e.g., less than 1 h) and are easily ignored when the temporal interval of the mobile phone location data is coarse (e.g., 1 h). Second, for the activity patterns derived by the state-of-the-art method, the proportions of “Leisure” activities are lower and the proportions of “Home” activities are higher than those derived by the proposed method. This finding tends to be due to the effect of the Monte Carlo simulation method in determining the final activity purposes since directly choosing the activity purpose with the highest probability tends to overestimate activity purposes with higher probabilities and underestimate those with lower probabilities.

## 5 Discussion

### 5.1 Sensitivity analysis of the weighting parameter for the spatial and temporal features

As Eq. (9) indicates, if *λ* is zero, only the temporal features are used to infer the activity purposes; if *λ* is one, only the spatial features are used to infer the activity purposes. Using the activity patterns derived from the travel survey data as the benchmark, we discuss the contributions of the spatial and temporal features by a sensitivity analysis of *λ*.

**Fig. 10:**
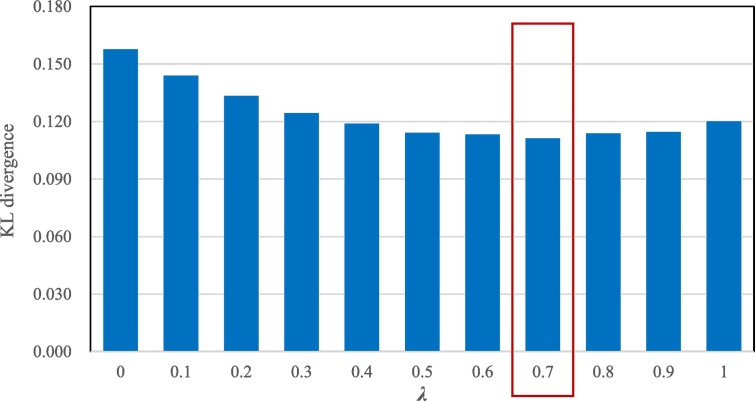
KL divergence values of the two activity patterns mined from the mobile phone location dataset and the travel survey dataset for different λ values.

Fig. 10 shows the KL divergence values between the activity patterns derived from the travel survey dataset and those derived from the mobile phone location dataset based on the proposed method for different values of *λ*. A smaller KL divergence value indicates better performance. It is obvious that the KL divergence values exhibit a valley shape.

The valley shape of the bins in Fig. 10 indicates that the spatial and temporal features working together can deliver a better result in activity purpose inference than using one of them alone. Specifically, when *λ* is 0.7, the proposed method achieves the best performance (*D*_*KL*_^*λ*=0.7^ = 0.111). If there is no further reference information, we recommend that *λ* could be set to 0.5 (the spatial and temporal features work equally for the outcomes) to obtain a reasonable outcome that is close to the best performance (*D*_*KL*_^*λ*=0.5^ = 0.111 vs. *D*_*KL*_^*λ*=0.7^ = 0.114) by taking both features into consideration.

Fig. 10 also suggests that if only one of the spatial or temporal features is available, the model performance is still acceptable, but using only spatial features to infer activity purposes is relatively more reliable. Specifically, the KL divergence *D*_*KL*_^*λ*=0.0^ = 0.158 for *λ* = 0.0 is larger than *D*_*KL*_^*λ*=1.0^ = 0.120 for *λ* = 1.0 (see Table A.1 in Appendix A for detailed information). Compared to the best performance of *D*_*KL*_^*λ*=0.7^ = 0.111, the KL divergence performance of the model using only the spatial features (*λ* = 1.0) decreases by less than 10%.

In terms of data availability, the above findings have great practical meaning. In the ICT age, spatial data about the built environment such as POIs have become increasingly accessible (Chen et al., 2020; Yue et al., 2017), but for many areas, data resources such as travel surveys that can produce the temporal features used in our method are not easily accessible due to data restrictions or the high cost of conducting a large-scale survey. Therefore, if the data condition is not good enough, finding a dataset that contains the spatial features tends to be easier and more productive than finding a dataset that contains temporal features. Our results suggest that the proposed method can offer a reasonable alternative to mining activity chains from mobile phone location data based on the spatial features data.

### 5.2 The robustness of the results by the size of the mobile phone location dataset

To test the robustness of the results by the data size, we randomly select subsets from the mobile phone location dataset with different sampling rates. Using the activity patterns derived from the entire dataset as the benchmark, we then test the performance of the results derived from the sub-datasets with different sampling rates via the KL divergence.

**Fig. 11:**
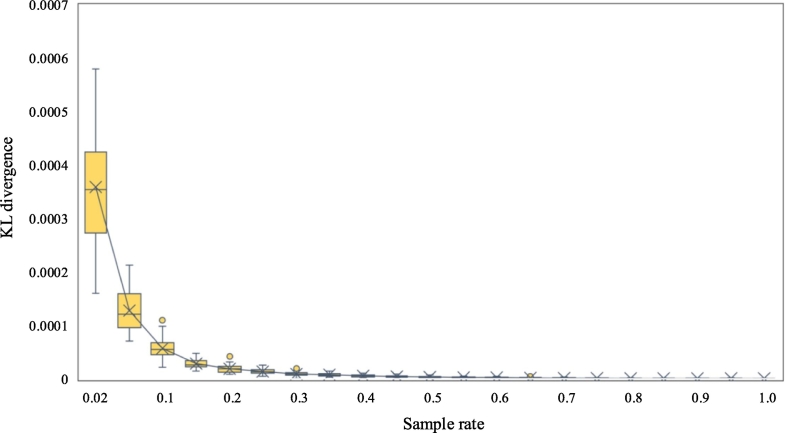
The performance of mining activity chains with different mobile phone location dataset sizes. The activity patterns derived from the entire dataset are used as the benchmark to calculate the KL divergence. Thirty percent of the users in the dataset corresponded to approximately 16.5% of the population of Shenzhen in 2012.

Specifically, we design the sampling rate series to include a sampling rate of 2% (the sampling rate of the Shenzhen travel survey data) and a range of sampling rates between 5% and 100% with a step size of 5%. Each sampling rate is randomly tested 50 times, and we use the mean KL divergence of the derived activity pattern distributions to evaluate the performance. The results are shown in Fig. 11.

We find that the KL divergence decreases with increasing sampling rate, and this finding implies that larger data sizes result in better performance for the activity chain inference in this study. However, the decrease in KL divergence caused by a 5% data volume increase converges to less than 0.3 when the sampling rate reaches 30% (see Table A.2 in Appendix A). This result suggests that 30% of the mobile phone location dataset is enough to generate the major activity patterns of all the users with similar performance to that of the whole dataset. Considering that the population of Shenzhen was 10.54 million in 2012 (Gazette of the People's Government of Shenzhen Municipality 2012) and that 30% of the users in the dataset used in this study is 1.74 million people, our results suggest that a sampling rate of 16.5% could generate a relatively robust outcome when inferring the main activity chains of a megacity such as Shenzhen.

In the big data era, many researchers want to use as much data as possible. Larger data sizes do seem to result in better outcomes (i.e., the results shown in Fig. 11). However, more data implies larger privacy concerns, higher sensitivity issues and lower processing efficiency, which make the datasets more difficult to access and process. Fig. 11 indicates an important insight: obtaining an outcome with reasonable performance may not require the whole dataset. This issue is related to the classic topic of “How big is big enough of the sample size?” in many applied statistics fields such as social science and education (e.g., Brunner, 2010; Chuah et al., 2006; Tanaka, 1987). However, this topic has seldom been discussed in previous human mobility studies. A better understanding of this topic enables us to make better use of related big trajectory datasets with different data conditions. For example, only a subset of the whole dataset (e.g., covering 20% of the population) can generate reliable outcomes. As a result, these will be less privacy concerns and higher data processing efficiency in practical applications.

It is important to note that the specific number of the lowest “sample size” varies across cities and research purposes. For example, 16.5% of the population could be enough to produce the major activity patterns of the users in Shenzhen. However, the specific number might change if the activity patterns of a special group of people (e.g., elderly people) are needed or the study area is changed to another city (e.g., Boston or Shanghai).

### 5.3 Effectiveness analysis of the proposed model and its implications

Our results show that the frequency distribution of major activity chain patterns and the temporal distribution of different activity purposes generally agree well with the patterns reported by the travel survey. Therefore, we conclude that the proposed model is effective. We think there are three major reasons that explain this effectiveness. (1) The stop identification algorithm we proposed is effective and is the foundation of this kind of model. An activity stop we identify comes with an activity location and activity time including the activity start time and duration. The spatial and temporal information corresponding to an activity stop is critical for inferring the activity type. Moreover, the order of stops forms the entire structure of an activity chain. (2) The POI characteristics can well represent people's activity types at an aggregate level. This is the social bond between people's activities and their built environment, representing the coupling constraints between individual activities and places as framed by time geography (Hägerstrand, 1970). (3) Although the represented populations of the travel survey and mobile phone users are not identical, the sampling bias does not significantly affect the activity transition probabilities with temporal dynamics. Therefore, the activity transition probabilities reported by the travel survey work well for the mobile phone users at an aggregate level.

In addition to the above reflections, the results about the effects of spatial and temporal features on the model performance deliver more implications. First, the spatial and temporal features working together can deliver a better result in activity purpose inference than using one of them alone. This finding is consistent with our common knowledge and previous theories. On the one hand, from the spatial perspective, there is a strong coupling constraint between people's activities and their built environment as suggested by time geography (Hägerstrand, 1970). For instance, people are likely to work if they are in workplaces, while people are likely to stay at home if they are in residential areas. As a result, at an aggregate level, the inference of activity type based on POI analysis will be useful. On the other hand, from the temporal perspective, most urban residents (especially commuters) have similar orders of weekday activities, which will generate some major transition patterns in the temporal dimension. Therefore, at an aggregate level, the temporal transition patterns will contribute to the inference of activity type. Overall, these two types of useful features will regulate each other and deliver a better result if they work together.

Second, the spatial features play a more important role than the temporal features in activity purpose inference. This finding suggests a stronger association with place for an activity than temporal association. Note that this is from an observation angle rather than a motivation angle. Moreover, technically, POI analysis based on each cell tower (i.e., there are approximately 6000 cell towers in this study) will offer more diversity in the probability distributions of activity types than the transition patterns based on each time interval (i.e., there are 24 time intervals in this study). The greater diversity offered by spatial features may be more in line with human behavior.

## 6 Conclusions

This study proposes a flexible method to mine human activity chains from large-scale mobile phone location data by integrating both the spatial and temporal features of daily activities.

Using a large-scale mobile phone location dataset, a POI dataset and a travel survey dataset for Shenzhen city, this study demonstrates the effectiveness of the proposed method for mining activity chains. Our results show that, in general, the frequency distribution of the major activity chain patterns and the temporal distribution of different activity purposes agree well with the patterns reported by a travel survey for Shenzhen. In addition, our results also show that a sampling rate of 16.5% of the total urban population for a mobile phone location dataset can deliver a reasonable and robust outcome of the major activity patterns, and this finding can contribute to big data research and practical applications.

Another interesting result shows that the spatial and temporal features working together can deliver a better result in activity purpose inference than using one of them alone. In addition, our results also suggest that using only the spatial features derived from the POI data to infer the activity purposes can be relatively more reliable than using only the temporal features derived from the travel survey data. Considering that POIs are often easily accessible, while travel surveys are more difficult to collect, this finding could be helpful in practice. Overall, the successful application of this kind of model needs a combination of big data and small data, consistent multidisciplinary research and synergistic cooperation among different social factors.

In future studies, the following two directions are worthy of analysis. First, mobile phone location datasets from other cities can be utilized to verify the generality of the proposed trip chain mining framework. Parameters such as the distance threshold and the weighting coefficient, which are included in this framework, can also be estimated by self-adaptive algorithms to determine the optimal solution. Second, mobile phone location data with a longer sampling period (i.e., more than one week) can be used to capture the periodic regularity of individuals' activity patterns. The effectiveness of inferring inter-day activity patterns could be estimated. Third, the accessibility of different POIs at different times of a day matters when inferring the activity purposes located in an area with a large mix of POI categories (i.e., downtown areas with shopping malls and office buildings in Shenzhen). This feature could be considered to improve the performance of activity purpose inference from mobile phone location data.

## 7 Takeaway for practice

Firstly, if an application involves high computing requirements or privacy concerns, it is critical to be aware of that not the whole dataset is required to achieve a reasonable outcome. We find that a sub dataset including users that account 16.5% of the population can provide a reasonable outcome of the major patterns of the activity chains.

Secondly, in terms of mining activity types from mobile phone location data, spatial features explicit stronger impacts than the temporal features. In this study, although the combination of the spatial and temporal features can generate a better outcome of inferring major activity chains, the spatial features alone can also generate a reasonable outcome. Therefore, when the datasets that can provide temporal activity patterns such as detailed travel survey are not available, the data sources involving spatial features such as POI datasets, which are more accessible can be used to produce acceptable outcomes in a general picture.

Third, short-lasting activities (e.g., “Leisure activities”) tends to be neglected in mobile phone locations data that usually have relative sparse temporal samplings. This kind of data should be carefully used to support official urban policy-makings such as leisure-oriented transport system design, leisure space planning and management and intervention for disease spread through leisure activities. Fully understanding of the shortcomings and corresponding impacts of this commonly-used data type is required before serious policies are made based on the derived analysis results.

## CRediT authorship contribution statement

**Ling Yin:** Conceptualization, Methodology, Writing - Reviewing and Editing, Funding acquisition.

**Nan Lin:** Methodology, Software, Writing - Original draft preparation, Visualization,

**Zhiyuan Zhao:** Conceptualization, Methodology, Visualization, Software, Writing - Original draft preparation, Writing - Reviewing and Editing, Validation, Funding acquisition.

## Declaration of competing interest

The authors declare that they have no known competing financial interests or personal relationships that could have appeared to influence the work reported in this paper.

## Figures and Tables

**Table A.1:** The probability distributions of the activity pattern for the proposed method for different parameter settings of *λ*.

Activity patterns	Mobile phone location data with different *λ*											Travel survey
	0	0.1	0.2	0.3	0.4	0.5	0.6	0.7	0.8	0.9	1	
HWH	72.78%	71.61%	70.41%	69.13%	67.93%	66.59%	65.23%	63.87%	62.46%	60.91%	59.27%	56.98%
HWHWH	6.46%	6.49%	6.51%	6.49%	6.51%	6.55%	6.53%	6.55%	6.57%	6.56%	6.62%	9.86%
HSH	5.71%	5.93%	6.17%	6.36%	6.58%	6.83%	7.08%	7.29%	7.52%	7.85%	8.10%	7.23%
HLH	3.92%	4.14%	4.37%	4.64%	4.87%	5.11%	5.34%	5.57%	5.81%	6.06%	6.32%	5.49%
HSHSH	0.49%	0.48%	0.49%	0.50%	0.51%	0.51%	0.51%	0.52%	0.52%	0.54%	0.52%	3.12%
HWLH	0.28%	0.41%	0.54%	0.67%	0.79%	0.92%	1.05%	1.19%	1.32%	1.46%	1.59%	1.80%
HWHLH	0.22%	0.21%	0.19%	0.18%	0.16%	0.15%	0.13%	0.12%	0.10%	0.09%	0.08%	1.69%
HOH	1.93%	2.13%	2.33%	2.55%	2.71%	2.90%	3.12%	3.31%	3.49%	3.64%	3.86%	1.58%
Other patterns	8.21%	8.60%	8.99%	9.48%	9.94%	10.44%	11.01%	11.58%	12.21%	12.89%	13.64%	12.25%

**Table A.2:** The detailed number of the KL divergence derived from the dataset with different volume in Fig. 11.

ID	Size	KL divergence (10^5^)^a^				*Delta*^b^	
		*Q1*	*Q2*	*Q3*	*Avg*	*Q2*	*Avg*
1	0.02	27.36	35.46	42.53	35.94	/	/
2	0.05	9.62	12.12	15.98	12.81	0.66	0.64
3	0.1	4.53	5.51	6.81	5.74	0.55	0.55
4	0.15	2.29	2.62	3.43	2.88	0.52	0.50
5	0.2	1.25	1.89	2.35	1.88	0.28	0.35
6	0.25	1.09	1.34	1.71	1.38	0.29	0.26
7	0.3	0.68	0.91	1.16	0.92	0.32	0.33
8	0.35	0.54	0.70	0.99	0.77	0.24	0.16
9	0.4	0.36	0.57	0.74	0.57	0.19	0.26
10	0.45	0.30	0.44	0.57	0.45	0.22	0.22
11	0.5	0.23	0.34	0.46	0.35	0.24	0.22
12	0.55	0.18	0.25	0.34	0.26	0.25	0.25
13	0.6	0.18	0.24	0.31	0.25	0.07	0.03
14	0.65	0.13	0.19	0.25	0.20	0.18	0.22
15	0.7	0.10	0.13	0.21	0.16	0.34	0.19
16	0.75	0.09	0.13	0.17	0.13	0.01	0.19
17	0.8	0.06	0.10	0.14	0.11	0.20	0.14
18	0.85	0.06	0.09	0.12	0.09	0.16	0.16
19	0.9	0.06	0.08	0.13	0.10	0.02	0.03
20	0.95	0.05	0.09	0.13	0.10	0.06	0.02
21	1	0.05	0.07	0.10	0.08	0.24	0.20

a The *Q*1, *Q*2 and *Q*3 indicates the first, second and third quartile, respectively, *Avg* indicates the average value.

b *Delta* is adopted to reflect the relative changes of Q2 and Avg when the data volume size changes by the step of 5% (also 0.05 in this table) according to the following equation: $\mathrm{Delta}\left(v_{i}\right)=\mathrm{abs}\left(v_{i}/v_{i-1}-1\right)$ where *i* indicates the number of the column ID and *v*_*i*_ indicate corresponding values of *Q*2 or *Avg*. The ***abs*** indicates the absolute function.

## References

[bb0010] Ahas R., Aasa A., Yuan Y., Raubal M., Smoreda Z., Liu Y., Zook M. (2015). Everyday space–time geographies: Using mobile phone-based sensor data to monitor urban activity in Harbin, Paris, and Tallinn. International Journal of Geographical Information Science.

[bb0015] Aleta A., Martín-Corral D., Pastore y Piontti A., Ajelli M., Litvinova M., Chinazzi M., Moreno Y. (2020). Modelling the impact of testing, contact tracing and household quarantine on second waves of COVID-19. Nature Human Behaviour.

[bb0020] Allen L.J.S., Brauer F., van den Driessche P., Wu J. (2008). An introduction to stochastic epidemic models. Mathematical epidemiology.

[bb0025] Bello I., Zoph B., Vasudevan V., Le Q.V. (2017). Neural optimizer search with reinforcement learning. In: International conference on machine learning. Presented at the international conference on machine learning.

[bb0030] Blondel V.D., Decuyper A., Krings G. (2015). A survey of results on mobile phone datasets analysis. EPJ Data Science.

[bb0035] Bowman J.L., Ben-Akiva M.E. (2001). Activity-based disaggregate travel demand model system with activity schedules. Transportation Research Part A: Policy and Practice.

[bb0040] Brunner L.A.K. (2010). “How big is big enough?”-Steve, big, and phallic masculinity in sex and the city. Feminist Media Studies.

[bb0045] Cao J., Li Q., Tu W., Wang F. (2019). Characterizing preferred motif choices and distance impacts. PLoS One.

[bb0050] Chen Y., Chen X., Liu Z., Li X. (2020). Understanding the spatial organization of urban functions based on co-location patterns mining: A comparative analysis for 25 Chinese cities. Cities.

[bb0055] Chen C., Ma J., Susilo Y., Liu Y., Wang M. (2016). The promises of big data and small data for travel behavior (aka human mobility) analysis. Transportation Research Part C: Emerging Technologies.

[bb0060] Chinazzi M., Davis J.T., Ajelli M., Gioannini C., Litvinova M., Merler S., Vespignani A. (2020). The effect of travel restrictions on the spread of the 2019 novel coronavirus (COVID-19) outbreak. Science.

[bb0065] Chuah S.C., Drasgow F., Luecht R. (2006). How big is big enough? Sample size requirements for CAST item parameter estimation. Applied Measurement in Education.

[bb0070] Çolak S., Alexander L.P., Alvim B.G., Mehndiratta S.R., González M.C. (2015). Analyzing cell phone location data for urban travel: Current methods, limitations and opportunities. Transportation Research Record: Journal of the Transportation Research Board.

[bb0075] Collia D.V., Sharp J., Giesbrecht L. (2003). The 2001 National Household Travel Survey: A look into the travel patterns of older Americans. Journal of Safety Research.

[bb0080] Deville P., Linard C., Martin S., Gilbert M., Stevens F.R., Gaughan A.E., Blondel V.D., Tatem A.J. (2014). Dynamic population mapping using mobile phone data. Proceedings of the National Academy of Sciences.

[bb0085] Diao M., Zhu Y., Ferreira J., Ratti C. (2016). Inferring individual daily activities from mobile phone traces: A Boston example. Environment and Planning B: Planning and Design.

[bb0090] Esztergár-Kiss D., Rózsa Z., Tettamanti T. (2017). Comparative analysis of test cases of the activity chain optimization method. Transportation Research Procedia.

[bb0095] Golledge R.G., Stimson R.J. (1997). Spatial behavior: A geographic perspective.

[bb5005] Gong L., Liu X., Wu L., Liu Y. (2016). Inferring trip purposes and uncovering travel patterns from taxi trajectory data. Cartography and Geographic Information Science.

[bb0100] Hägerstrand T. (1970). What about people in regional science?. Papers in Regional Science.

[bb0105] Hartgen D.T., San Jose E. (2009). Costs and trip rates of recent household travel surveys.

[bb0110] Horn C., Klampfl S., Cik M., Reiter T. (2014). Detecting outliers in cell phone data: Correcting trajectories to improve traffic modeling. Transportation Research Record: Journal of the Transportation Research Board.

[bb0115] Iovan C., Olteanu-Raimond A.-M., Couronné T., Smoreda Z., Vandenbroucke D., Bucher B., Crompvoets J. (2013). Moving and calling: Mobile phone data quality measurements and spatiotemporal uncertainty in human mobility studies. Geographic Information Science at the Heart of Europe.

[bb0120] Kitamura R. (1988). An evaluation of activity-based travel analysis. Transportation.

[bb0125] Kullback S. (1997). Information theory and statistics.

[bb0130] Martin W.A., McGuckin N.A. (1998). Travel estimation techniques for urban planning.

[bb0135] McDonald N.C. (2008). Critical factors for active transportation to school among low-income and minority students: Evidence from the 2001 National Household Travel Survey. American Journal of Preventive Medicine.

[bb0005] McGraw-Hill Concise Dictionary of Modern Medicine S.v. “contact rate”. https://medical-dictionary.thefreedictionary.com/contact+rate.

[bb0140] Meloni I., Bez M., Spissu E. (2009). Activity-based model of women’s activity–travel patterns. Transportation Research Record.

[bb0145] Olsen P.A., Dharanipragada S. (2003). An efficient integrated gender detection scheme and time mediated averaging of gender dependent acoustic models.

[bb0150] Ratti C., Pulselli R.M., Williams S., Frenchman D. (2006). Mobile landscapes: Using location data from cell phones for urban analysis. Environment and Planning B: Planning and Design.

[bb0155] Saravanan M., Karthikeyan P., Arathi A., Kiruthika M., Suganya S. (2013). Mobile agent-based approach for modeling the epidemics of communicable diseases. Proceedings of the 2013 IEEE/ACM International Conference on Advances in Social Networks Analysis and Mining.

[bb0160] Tanaka J.S. (1987). “How big is big enough?”: Sample size and goodness of fit in structural equation models with latent variables. Child Development.

[bb0165] Tatem A.J., Huang Z., Narib C., Kumar U., Kandula D., Pindolia D.K., Smith D.L., Cohen J.M., Graupe B., Uusiku P., Lourenço C. (2014). Integrating rapid risk mapping and mobile phone call record data for strategic malaria elimination planning. Malaria Journal.

[bb0170] Tu W., Cao J., Yue Y., Shaw S.-L., Zhou M., Wang Z., Li Q. (2017). Coupling mobile phone and social media data: A new approach to understanding urban functions and diurnal patterns. International Journal of Geographical Information Science.

[bb0175] Widhalm P., Yang Y., Ulm M., Athavale S., González M.C. (2015). Discovering urban activity patterns in cell phone data. Transportation.

[bb0180] Xu Y., Shaw S.-L., Zhao Z., Yin L., Lu F., Chen J., Li Q. (2016). Another tale of two cities: Understanding human activity space using actively tracked cellphone location data. Annals of the American Association of Geographers.

[bb0185] Yang X., Fang Z., Yin L., Li J., Lu S., Zhao Z. (2019). Revealing the relationship of human convergence–divergence patterns and land use: A case study on Shenzhen City, China. Cities.

[bb0190] Yang X., Zhao Z., Lu S. (2016). Exploring spatial-temporal patterns of urban human mobility hotspots. Sustainability.

[bb5000] Yin L., Chen J., Zhang H., Yang Z., Wan Q., Ning L., Yu Q. (2019). Improving emergency evacuation planning with mobile phone location data. Environment and Planning B: Urban Analytics and City Science.

[bb0200] Yin L., Lin N., Song X., Mei S., Shaw S.-L., Fang Z., Li Q., Li Y., Mao L. (2019). Space-time personalized short message service (SMS) for infectious disease control – Policies for precise public health. Applied Geography.

[bb0205] Yin M., Sheehan M., Feygin S., Paiement J., Pozdnoukhov A. (2018). A generative model of urban activities from cellular data. IEEE Transactions on Intelligent Transportation Systems.

[bb0210] Yue Y., Zhuang Y., Yeh A.G.O., Xie J.-Y., Ma C.-L., Li Q.-Q. (2017). Measurements of POI-based mixed use and their relationships with neighbourhood vibrancy. International Journal of Geographical Information Science.

[bb0215] Zhao Z., Shaw S.-L., Yin L., Fang Z., Yang X., Zhang F., Wu S. (2019). The effect of temporal sampling intervals on typical human mobility indicators obtained from mobile phone location data. International Journal of Geographical Information Science.

[bb0220] Zhao Z., Yin L., Shaw S.-L., Fang Z., Yang X., Zhang F. (2018). Identifying stops from mobile phone location data by introducing uncertain segments. Transactions in GIS.

[bb0225] Zhou Y., Xu R., Hu D., Yue Y., Li Q., Xia J. (2020). Effects of human mobility restrictions on the spread of COVID-19 in Shenzhen, China: A modelling study using mobile phone data. The Lancet Digital Health.

